# Allied health care in the early stages of the COVID-19 pandemic: A qualitative study on the perceptions of non-hospitalized patients and allied health professionals

**DOI:** 10.1371/journal.pone.0341308

**Published:** 2026-01-23

**Authors:** Carla Agasi-Idenburg, Anja de Kruif, Amber Ronteltap, Sonja van Oers, Ton Satink, Marian de van der Schueren, Cindy Veenhof

**Affiliations:** 1 Research Group Innovation of Movement Care, University of Applied Sciences Utrecht, Utrecht, The Netherlands; 2 Department of Nutrition, Dietetics and Lifestyle, HAN University of Applied Sciences, Nijmegen, The Netherlands; 3 Division of Human Nutrition and Health, Wageningen University and Research, Wageningen, The Netherlands; 4 Department of Rehabilitation, Physical Therapy Science and Sport, Brain Center, University Medical Center Utrecht, Utrecht University, Utrecht, The Netherlands; Queensland University of Technology - QUT: Queensland University of Technology, AUSTRALIA

## Abstract

**Purpose:**

Worldwide, the COVID-19 pandemic disrupted access to primary care and rehabilitation services, leaving many non-hospitalized patients with persistent symptoms unsure where to seek support. Primary allied health care (pAHP) became an important source of guidance and rehabilitation in several countries. This qualitative study explored how pAHP was accessed, experienced, and delivered in the Netherlands during the early stages of the pandemic, focusing on non-hospitalized patients with lingering symptoms following SARS-CoV-2 infection and the allied health professionals who supported them.

**Methods:**

A qualitative study was conducted in the Netherlands between January and May 2021. Semi-structured interviews were held with 11 non-hospitalized patients and 30 primary allied health care professionals across five disciplines. Participants reflected on their experiences with primary allied health care during the first year of the pandemic. Data were analyzed using inductive thematic analysis.

**Results:**

Three main themes described this period: (1) navigating uncertainty in the absence of guidelines and knowledge about the disease; (2) adapting care, balancing treatment content and delivery; and (3) forging connections, including interprofessional collaboration and organization in primary care. Patients generally reported positive experiences and valued feeling heard and supported, while professionals described adaptive strategies and organizational challenges.

**Conclusions:**

Primary allied healthcare played a valued and adaptive role for non-hospitalized patients during the early pandemic. These findings underscore the importance of acknowledging patients’ symptoms, strengthening interprofessional collaboration, and supporting flexible, evidence-informed care strategies to prepare primary care systems for future health crises.

## Introduction

In December 2019, the first case of the coronavirus (SARS-CoV-2), the virus that causes COVID-19 disease, was registered in China [1]. Within a month, the first cases of SARS-CoV-2 infection were reported in Europe [2,3]. By the end of February 2020, the National Institute for Public Health and the Environment Netherlands reported the first patient in the Netherlands [4]. The pandemic started in the South of the Netherlands. The scale at which care had to be delivered shortly thereafter highlights the importance of evaluating our response strategies.

While initial healthcare and academic research primarily focused on hospitalized COVID-19 patients, there was also a growing need for a substantial number of individuals also required primary care management of individuals experiencing ongoing symptoms after managing their acute infection at home but continuing to experience persistent symptoms after SARS-CoV-2 infection. In the early stages of the pandemic, this cohort mostly included patients whose infection developed less acutely and were managed at home, as well as some who were discharged from hospital.

In the Netherlands, many such patients were referred by General Practitioners (GPs) or medical specialists to primary allied health care professionals (pAHPs) with the aim of facilitating their recuperation [5]. We use pAHPs to denote dietitians (DTs), exercise therapists (ETs), occupational therapists (OTs), physiotherapists (PTs), and speech and language therapists (SLs) working in primary care; “primary allied health care” refers to the services they provide in this setting. including dietitians (DT), exercise therapists (ET), occupational therapists (OT), physiotherapists (PT), and speech and language therapists (SL), Meanwhile, at the onset of the pandemic, primary allied health care services faced substantial operational challenges, including government-mandated closures, restrictions on in-person consultations, and a rapid shift toward remote care [6–8].

For this newly emerging patient group, there was very limited evidence or guidance on the appropriate form, content, and organization of allied health care [9]. To better understand this phase and improve future responses, it is important to explore the experiences of non-hospitalized patients with persistent post-COVID-19 symptoms and the perspectives of the primary allied health care professionals who treated them. These insights can contribute to the development of more effective healthcare strategies in future health crises.

Therefore, this study explores how non-hospitalized patients and pAHPs in the Netherlands access, experience and deliver primary allied health care for persistent symptoms following SARS-CoV-2 infection during the early stages of the pandemic?

## Methods

### Research design

This qualitative study is part of the Dutch ParaCov study [9], a national prospective cohort study designed to evaluate the longitudinal recovery trajectories of patients who received primary allied health care (pAHP) for persistent symptoms following COVID-19. The ParaCov study comprises both quantitative and qualitative components.

The current paper presents findings from the qualitative component of the ParaCov study which was grounded in a phenomenological approach and guided by a constructivist research paradigm. This approach was chosen to explore how individuals experienced primary allied health care during the early stages of the COVID-19 pandemic. The study specifically focused on the perspectives of patients who remained at home during their illness (non-hospitalized patients) and the primary allied health care professionals who supported them, with the aim of understanding how meaning emerged through their interactions and reflections.

The study was guided by a constructivist research paradigm, acknowledging that experiences of illness and care are co-constructed through interactions between individuals and their social, professional, and organizational environments. This paradigm informed data collection and analysis by emphasizing interpretation, contextual meaning, and the existence of multiple perspectives within the dataset.

### Study design

This qualitative study was designed to explore how primary allied health care was accessed, experienced, and delivered during the early stages of the COVID-19 pandemic (March to December 2020). The research focused on two key stakeholder groups: patients who remained at home during their illness and received primary allied health care, and the primary allied health care professionals (pAHPs) who supported them. By including both perspectives, the study aimed to gain a deeper understanding of the organization, content, and perceived value of pAHP in the primary care setting. The qualitative design enabled us to examine how patients engaged with allied health services and how professionals adapted their practices to support patients with post-COVID-19 symptoms in a rapidly changing healthcare landscape. Semi-structured interviews with both patients and pAHPs allowed for an in-depth exploration of how individuals navigated and interpreted primary allied health care during this period. This flexible interview format enabled participants to articulate in their own words while offering space to probe emerging topics shaped by uncertainties and evolving context of the early pandemic.

### Participants and sampling

Participants were recruited between January 9 and April 21, 2021, and all had to be involved in primary allied health care during the first year of the Covid-19 pandemic, either as patients receiving care or as allied health professionals providing it.

Originally, we included 25 patients across three cohorts, (patients admitted to an intensive care unit, those hospitalized on a COVID-19 ward, and those managed at home), to ensure sufficient variation.. However, presenting data from all three cohorts alongside the professional sample proved beyond the scope of a single paper. Following external reviewer feedback that combining multiple patient cohorts with the professional sample risked diluting coherence of design and interpretation, we refined the sampling frame to non-hospitalized patients receiving primary allied health care and to pAHPs, thereby aligning sampling more closely with the study aim and maintaining analytic focus.

We purposefully selected 11 patients who had remained at home throughout their illness and received primary allied health care. To ensure maximum variation This subgroup was chosen to allow for a focused exploration of primary care-based support during COVID-19. To ensure maximum variation, we aimed to include patients who differed in gender, level of education (both high and low), and geographic distribution across the Netherlands, with particular attention to the southern areas most affected during the initial phase of the COVID-19 outbreak. Patients were recruited through multiple channels: the Lung Foundation Netherlands distributed a study invitation via its newsletter; a regional hospital identified individuals with a documented SARS‑CoV‑2 infection in its records and mailed them an invitation letter; and primary allied health care practices in our professional networks informed eligible patients about the study. Snowball sampling was also used to enhance recruitment. Eligible participants were ≥18 years old, experienced activity limitations and/or participation restrictions due to COVID-19 and received allied health care by at least one pAHP in the primary care setting between March and December 2020. Patients receiving palliative care were excluded because their access, experience, and care delivery were likely to differ from those managed with curative intent, hence not aligning with our research question on recovery-focused primary allied health care. This exclusion also prevented placing undue burden on individuals receiving end-of-life care.

We adopted purposeful sampling to maximize information power, defined as the richness and relevance of data in relation to the aim, rather than targeting a numerical threshold or invoking ‘saturation’. Given the focused aim, specificity of the sample, established qualitative orientation (phenomenology/constructivism), high-quality interviews, and reflective thematic analytic strategy, 11 patient interviews were considered sufficient [10,11].

In addition, 30 pAHPs were included in the study. Participants were recruited through their respective Dutch professional associations and the authors’ professional networks. Specifically, the professional associations collaborating in the Consortium (Dutch society of speech therapy and phoniatrics, Dutch society of Dieticians, Occupational therapy Netherlands, Royal Society of physical therapy, Society of exercise therapists Cesar and Mensendieck), published a brief call for participation in their newsletters inviting pAHPs to take part. To ensure a broad range of perspectives, we purposefully selected six professionals from each of the five main allied health professions active in primary care: dietitians, exercise therapists, occupational therapists, physiotherapists, and speech and language therapists. Sampling aimed for variation in gender, years of experience, and geographic distribution across the Netherlands. All participating pAHPs worked in the primary care setting and had treated at least three patients with symptoms following SARS-CoV-2 infection in 2020.

### Data collection

Interviews were conducted by five members of the qualitative research team (four female, one male), all of whom had backgrounds in both healthcare and qualitative research (Carla Agasi-Idenburg, Anja de Kruif, Amber Ronteltap, Sonja van Oers, Ton Satink). The research team consisted of professionals with diverse backgrounds in healthcare, including a former physiotherapist and clinical health scientist, a former nurse and senior qualitative researcher, a nutritional scientist and a lifestyle coach, a former dietitian and researcher, and an former occupational therapist and professor. Prior to data collection, the interview guides were reviewed and piloted by two expert patients (individuals with lived experience of COVID disease recruited by the Lung Association) and three pAHPs (identified via professional associations). Minor wording and sequencing adjustments were made accordingly. See S1 Appendix and S2 Appendix for interview guides. Interviews were scheduled at participant-convenient times and designed to last approximately 60 minutes. All interviews were audio-recorded, and researchers kept contemporaneous field notes capturing salient context, verbal and non-verbal cues, and key points.

### Data analysis

Atlas.ti 9.0 software supported data management. Analysis followed Braun and Clarke’s six-phase thematic approach, underpinned by a constructivist lens [11]. A constant comparison approach [12] was also used to support the identification of patterns across narratives and to refine interpretations during the analytic process.

This multidisciplinary composition of the research team enriched the interpretation of the data but also required careful attention to potential bias. Throughout both data collection and analysis, the researchers employed reflexivity and bracketing techniques to minimize the influence of their own professional perspectives. By continuously reflecting on their assumptions and documenting their positionality, the team aimed to ensure that interpretations remained grounded in the participants’ narratives rather than shaped by prior knowledge or expectations. As analysis proceeded concurrently with data collection, emerging topic summaries, patterns, and insights were iteratively compared within and across participant accounts, informing the focus of subsequent interviews [13]. Following this initial calibration, the remaining interviews within this participant group were individually coded, drawing on and further developing the emerging topic summaries. Theme reviewing, defining and naming themes were conducted in the big study group of the five researchers (Carla Agasi-Idenburg, Anja de Kruif, Amber Ronteltap, Sonja van Oers, Ton Satink). Dependability was addressed by maintaining a detailed audit trail of the research process, including the development of the interview guide, the transcription process, and decisions on theme development.

### Ethics statement

This study was conducted in accordance with the ethical standards of the Declaration of Helsinki and was approved by the Human Ethics Research Committee of the HAN University of Applied Sciences (reference number ECO 301.1/21). All participants received written and/or verbal information about the study and provided informed consent prior to participation. The ethics committee approved the use of both written and verbal consent procedures. Participants were explicitly informed of their right to withdraw from the study at any time without consequence. Participants did not receive financial or other incentives for their participation.

## Results

A total of 41 participants were included in this study: 11 patients who remained at home during their COVID-19 illness and received primary allied health care, and 30 pAHPs, six from each of the five different disciplines.

### Patients (Pt)

Our study included eleven patients: five from the south, most of whom were healthcare professionals themselves, and six from other regions. See Table 1 for full details. The majority had contracted the virus at work. Four patients were from the central region, one from the western region, and one from the eastern region. This geographic distribution reflects the early spread of the virus in the Netherlands: the pandemic began in the south, leading to higher infection rates and earlier demand for care in that region, while infection rates remained relatively low in the east and north during the initial phase of the pandemic [13]. The patient sample was diverse in terms of gender, educational background.

**Table 1:** Characteristics of population.

Characteristic	Category	Non-hospitalized patients (n = 11)	Primary Allied Health Professionals (n = 30)
Age, mean (range)		43 (26-71)	38 (24-62)^‡‡^
Sex, n (%)	Male	2 (18%)	3 (10%)
	Female	9 (82%)	27 (90%)
Illness severity subgroup^‡^, n (%)	Moderate, severe	a = 8 (72%)	–
	Indication admission	b = 2 (18%)	
	Inpatient rehabilitation	c = 1 (9%)	
Professionals background patients, n (%)	pAHP*	2 (18%)	–
	Other healthcare background	3 (27%)	
	No healthcare background	6 (64%)	
pAHP Professional Role, n (%)	Dietitians	–	6 (17%)
	Exercise Therapists	–	6 (17%)
	Occupational Therapists	–	6 (17%)
	Physiotherapists	–	6 (17%)
	Speech and language Therapists	–	6 (17%)

**Explanation of subgroups and symbols**

^‡^Subgroups were defined as:

a) Moderate/Severe illness, no admission indication, stayed at home

b) Severely ill, indication for admission but stayed at home

c) Critically/severely ill, received inpatient rehabilitation (including rehabilitation center or nursing home)

*pAHP = primary care allied health professional

^‡‡^The reported age of pAHP was estimated based on their years of work experience, assuming a typical starting age and duration of professional training for Dutch AHP bachelor programs.

### Primary care allied health care professionals (pAHPs)

The sample was composed to reflect variation in demographic and professional characteristics. Although the aim was to include pAHPs who had treated at least three patients with post-COVID-19 symptoms, one exercise therapist was included who had treated only one patient, due to limited referrals in the early phase of the pandemic. We included 30 pAHPs, 6 participants across five disciplines, with a predominance of female participants and significant work experience, reflecting a balanced representation in terms of residence, work setting, and urbanization. See Table 1 for full details.

Interviews lasted between 30 and 74 minutes. One interview was conducted in two sessions across two days due to the participant’s limited energy. The analysis resulted in three overarching themes that capture the key patterns of meaning across the participant groups. These themes reflect shared and divergent experiences, and provide insight into the central issues raised by participants. Each theme is presented below with illustrative quotes to highlight the depth and nuance of participants’ perspectives.

### Theme 1 – Navigating the unknown: the impact of early pandemic uncertainty on primary allied health care provision and patient engagement

Early Stage COVID-19 in its early stages presented a landscape of profound uncertainty, significantly impacting both the provision and reception of primary allied health care. This overarching theme, “Navigating the unknown: the impact of early pandemic uncertainty on primary allied health care provision and patient engagement,” captures the inherent professional insecurity felt by pAHPs and referring physicians, alongside the complex emotional and practical challenges faced by patients as they sought care for an unfamiliar illness.

For primary allied health care professionals, the lack of established knowledge about COVID-19 created substantial professional insecurity. They were confronted with treating an “unknown disease,” leading to a perceived risk to themselves, their vulnerable family members, and other patients.

An occupational therapist (OT3) and private practice owner told us: *“What I found very intense is that I, had to make choices for my staff from a safe position, who might have to go out…, one person who was mainly afraid of infecting* [patients] *and another who was especially afraid because, they have a vulnerable family member working in a facility, a sister, vulnerable parents, um, and that ethical dilemmas arise, which was also a bit challenging. Am I going to impose something on someone like, you, you’re so afraid, you stay home* [laughs]*, um, and the one* [occupational therapist] *who wasn’t so afraid just went out, you know, hoping for the best. So, I found that very intense.*

This necessitated a reliance on their existing experience with comparable conditions like Q fever, chronic obstructive pulmonary disease (COPD), or chronic fatigue syndrome, as specific guidelines were non-existent.

A physical therapist (PT6) said: *“it was really more trial and error, of course, having done the courses from the chronic care network, etc. But that was nothing more than information provision, you know. Uh. And there wasn’t much more at the time. Um. So we just sort of copied and pasted it from the COPD rehabilitation program we have..”.*

Several patients (Pt), particularly in the early phase of the pandemic, had professional backgrounds in healthcare and actively shared information with their primary allied health care professionals regarding the management of SARS-CoV-2-related symptoms and side effects.

Pt8: *My physiotherapist stays informed, and she recently attended a webinar, I believe from the University of Applied Sciences Utrecht, … and now and then I pass information on to her.*

Despite these considerable uncertainties and risks, pAHPs demonstrated strong professional dedication, proactively seeking out limited scientific evidence and information. There was an immediate demand for timely guidance from professional associations, with some pAHPs expressing a preference for rapid dissemination of available knowledge through expedient methods such as webinars or factsheets, rather than waiting for comprehensive guidelines. This highlights the adaptive, yet insecure, environment in which allied health care was delivered. Some less experienced healthcare providers contacted more experienced colleagues for advice, further demonstrating the collaborative effort to mitigate uncertainty in care provision. Other pAHPs chose to work independently.

For patients (Pts) receiving primary allied health care, the uncertainty surrounding their SARS-CoV-2 infection and its recovery trajectory had a profound impact on their engagement with the healthcare system. Patients experienced fear, isolation (especially those managing symptoms at home without physical contact with a GP), and a lack of clarity regarding the disease’s progression and contagiousness, which often led to prolonged self-isolation.

Pt13:”*And no one has been inside (the house) during those three weeks, right? At that time, little was known (about the disease). Yes, if the general practitioner didn’t need to come, he wouldn’t come……. Well, we fortunately have a different perspective now, because it was quite lonely.”*

This pervasive uncertainty, coupled with the perception that General Practitioners (GPs) were overwhelmed with acute cases, sometimes resulted in patients delaying asking for help or seeking allied health care without a direct referral. While general patient experiences of acute illness are acknowledged as the backdrop, the focus here is on how this fundamental uncertainty about their condition shaped their need for and interaction with primary allied health care and their appreciation for pAHPs who offered recognition and tailored support amidst the unknown.

Pt5: *“Mainly the recognition that it was a real problem. That was very important in the beginning.., because back then, everyone was saying, including all the general practitioners and such, it’s all in your head. It’s not physical. Well, in the first few months I could still somewhat understand that, but at a certain point you think, this really isn’t just in my head. Something is going on here. At the physio there were several people who had the same thing. At least, a similar picture, and that it was a kind of acknowledgment. That was very important to me at the time. That really helped me”*

Pt4: *“The allied care professionals, within the framework of ‘we don’t know’, they still look at ‘what is possible’ and how they can improve the patients quality of life and how they can, yes, really improve the level of functioning as well”*

### Theme 2 – Adapting care: the evolving content and delivery of primary allied health treatment

Building on the initial uncertainty surrounding COVID-19, primary allied health care professionals (pAHPs) and referring physicians quickly embarked on a critical “search for balance” regarding the content and form of treatment for patients with persistent post-COVID-19 complaints. This theme highlights the dynamic adaptation of allied health care as knowledge evolved and practical constraints necessitated new approaches.

In terms of treatment content, pAHPs initially focused on applying their existing expertise to the novel symptoms. Physiotherapists, for example, began with strength, conditioning, and breathing exercises, but soon discovered that over-exertion could be counterproductive, leading to patient relapses. This forced a critical shift towards teaching patients to “restrain themselves” and focusing on the crucial balance between “load and capacity”.

ET5: *“But what I found really bizarre about that, she* [the patient] *had to do a fitness test in the hospital at some point. And then she had to keep going, going, going, so she had to push way past her limit. And after that, she deteriorated so badly. She had finally built up to the point where she could walk outside for about five minutes. And she crashed so hard that she was almost bedridden again all day. And that really shocked me. I thought, okay, a stress test is all well and good, but only up to your limit. Because apparently, if for whatever reason you go way beyond it, it really does something to your body. And sure, maybe in her case it was a coincidence, but come on, you’re 18. Seriously.”*

Dietitians concentrated on optimizing protein intake and balancing energy intake with physical activity. Though they noted taste and smell problems, which they could have addressed, these problems were not typically treated in the early phase. Occupational therapists centered on helping patients balance daily activities, managing limitations, and planning their day and week. Physical therapist mainly focused on increasing muscle strength and cardiovascular exercise capacity. Speech and language therapists focused on breathing patterns, muscle tension, and respiratory muscle strength.

Due to a lack of specific guidelines for COVID-19, the approach often involved “trial and error,” with pAHPs “copying and pasting” from existing rehabilitation programs for conditions like COPD.

DT2:” *I found it, you know, regarding nutrition and COVID, I found it very interesting because it’s completely new, so it’s not comparable to anything else. There are no guidelines at all, and you definitely have to, well, when I started in March, I really had to figure out a lot, you know, how to do it, how to get the information, how do other dietitians do it? Do you look at the hospitals where, actually, they were a bit ahead*. “

The severe breathlessness, pain, and “enormous muscle acidosis” experienced by patients required a careful, adapted approach that recognized their unique physiological responses to even simple activities.

PT3: *“I have a very clear picture of the immense breathlessness with which people came to us, a very distinct picture of the shortness of breath, um... not controllable. So, whereas with lung diseases you can dose or control, that was actually not possible with this shortness of breath. And yes, a huge range of complaints of thoracic pain, pain in the throat radiating, if they did even a little too much, headaches, or for example, concentration problems,……, or word-finding difficulties, a very extensive disarray, and one of the symptoms at the beginning was enormous muscle acidosis. People did something and really got enormous pain in the muscles, so they also simply could not recover for a while, that lasted a long time, and also could not exercise well”*

The majority of patients expressed great appreciation for the care they received from pAHPs, valuing the recognition of their complaints. Patients found it “very helpful” to be able to “spar” with their pAHPs, analyzing their activities, energy levels, and setbacks, even though many continued to experience persistent symptoms despite receiving care.

Pt7: “*I think they’re worth their weight in gold, really. The physical therapist and the occupational therapist. Discussing ‘what did you do and what didn’t you do. And what were the consequences, and how did it happen if you had a setback or a relapse, and where did the consequences come from?’ Analyzing that together, both with the physical therapist about exercising and with the occupational therapist about energy expenditure. Hmm yes, I’m also quite analytical myself, but being able to spar with someone about it, I found that very helpful”*

Pt8: *“Of course, a healthy lifestyle, physiotherapy, speech therapy, all of that helps quite a bit… but it’s really time that does the healing, and you especially need to make sure you’re not too tense. That’s logical, right?”*

However, some patients felt a greater need for mental or psychological support.

Pt13: “*You’re really confronted with your limitations. At least, I am. I’m speaking for myself, how I experience it. You’re constantly fighting because you want more… The occupational therapist teaches you how to pace yourself, and the psychologist looks with you, like: “Hey, why do you want all that? She also holds up a mirror to you, right, like: ‘What went well in the past year”*

Pt20: *Then I… I have an external coach. That’s a luxury, really. In my position, I have an external coach who helps me. So they help me make strategic decisions. I reached out to them in December, like, I need you to help me with setting boundaries for myself. So that’s not an allied health professional, but still.. You could say it’s psychological support or whatever you want to call it, but it’s someone who just says to you, ‘Today you’re going to do this.’ Or, ‘I notice this isn’t working well. How are we going to handle that?’ So I did that again, and it was really nice to just talk to someone and ask, ‘How would you handle this?’“*

The form of treatment also underwent rapid transformation due to government lockdown measures. pAHPs were initially compelled to switch to remote care via phone calls, video calls, and apps. This new delivery method offered advantages like time saving and convenience for patients with limited energy or those preferring to avoid in-person contact.

However, pAHPs found it challenging to conduct physical examinations or observe patient movements effectively without in-person contact, stating,

ET2: “*It’s nice to be able to offer guidance through video calls, but for my specific work, it’s not feasible. Being able to observe breathing, posture, muscle tension or relaxation, you can’t see that well enough on a computer or laptop*”.

OT4: “*Video calling, for me… Yes, I’ve done it before, especially during the first lockdown, but that had nothing to do with COVID. I find video calling awful, because—you know, I always say you need to be able to feel people a bit. Feel where the problem really lies, and that’s—people are much more reserved over the phone than when you’re just with them in person. So I feel like you don’t uncover nearly all the problems that way*.”

Not all patients had the ability to use a computer, which what complicated the matter, advice had to be given over the telephone. One speech and language therapist highlighted the difficulty in providing advice without visual assessment, such as for swallowing issues.

SL2: “*When I see someone, I know very well how to act. I really need that. I think: well, I can’t go there, but I am curious how I can give good swallowing advice if I don’t see someone. I need to see how someone chews and swallows. If I say ‘that gentleman can’t eat bread’, and if I haven’t seen it, I really find it difficult to give an advise*”.

One patient shared, they appreciated not having to travel.

Pt8: *Video calls.. with speech therapy it is possible, that has happened before, like when I said: my son had the care and I didn’t have the energy to bike, then we did it via video call, with speech therapy. Physical therapy is a bit more difficult.“*

### Theme 3 – Forging connections: The organization and collaboration in primary allied health care

In the context of the pervasive uncertainty (Theme 1) and evolving treatment approaches (Theme 2), the organization of primary allied health care and the critical role of collaboration emerged as a significant theme. The initial chaos of the pandemic strained existing healthcare structures, necessitating rapid adjustments in referral pathways and inter-professional communication.

General Practitioners (GPs), traditionally the gatekeepers of primary care in the Netherlands, were “overwhelmed by the number of extremely sick patients” in the early pandemic, particularly in affected regions. This led to a perceived lack of care coordination for patients managing COVID-19 at home. Referring physicians sometimes lacked information about the specific services pAHPs could offer and found it easier to collaborate with pAHPs they had pre-existing relationships with, mostly physical therapists. While GPs and medical specialists predominantly referred patients to physiotherapists, other pAHPs expressed concern about the lack of referrals to their specialties. The role of coordinating primary health care was often informally delegated, predominantly to physiotherapists, leading to varied experiences and sometimes delaying referrals to other pAHPs, thereby threatening care accessibility.

Patients experienced diverse referral pathways to pAHPs. They often found it difficult to ask for help, fearing GPs were too busy with acute cases, leading them to delay seeking care or even directly accessing pAHPs without a referral.

Pt10: *When I look at, uhm… for example, the childcare workers at my son’s daycare who also had COVID, some of them are still not back at work…. I’ve told them many times to go do recovery care. You also notice a kind of hesitation, or maybe people feel their symptoms aren’t severe enough for that. Yes, I do think that can be a huge barrier, because a certain process has to start, of realizing that the symptoms are long-lasting. That also requires a kind of, yes,..an insight from someone. That takes time”.*

Some patients who requested a referral noted a delay by their GP.

Despite these initial organizational hurdles, multidisciplinary primary allied health care treatment was strongly advocated, and collaboration was generally perceived as both necessary and beneficial by pAHPs, believing it led to efficient treatment and communication.

SL2: *“And if something stands out for this target group, it is often a lack of energy. That’s what we try to address in a multidisciplinary way. Every few weeks, we have such a meeting. For example, occupational therapists carefully assess ‘what someone can do and how is the demand on them at home.’ You also have to consider that someone may not be able to come every week and make choices accordingly. So, you need to have that broad perspective, which is important. And working with the physiotherapist on fitness, understanding what is possible and what that means for them, or what the physiotherapist can contribute, so that we overlap and speak the same language. It’s important for that multidisciplinary aspect, so that the client doesn’t receive different advice from each of us*.”

However, establishing these collaborations was challenging during the “turbulent times of the initial lockdowns”. Pre-existing multidisciplinary teams, such as those for COPD or Q-fever, facilitated smoother coordination and collaboration.

OT3: “ *“We had weeks during those first crisis weeks.. where we had… with one health center I’m part of, with several GP practices, a physiotherapy practice, a psychologist, speech therapist, uh, dietitian, we had a kind of crisis meeting every week. How are we doing things now? And what are you allowed to do now? And what are you running into, and what do we know now, and what are the current guidelines?”*

Healthcare professionals who lacked such networks found forming new collaborative networks too time-consuming.

ET3: *“Yes, but I was already doing that [working multidisciplinary] because I’m involved in Parkinson’s care. I think that as a therapist you’re really not doing a good job if you don’t have a multidisciplinary network around you. You can’t do it alone. No, it absolutely has added value. It’s necessary too. And I know plenty of exercise therapists who really work on their own little island. That just doesn’t work.”*

Concerns were also raised by both patients and healthcare professionals about patients already burdened by multiple care professionals, indicating a need for streamlined coordination.

Pt8: “*At a certain point, what becomes quite challenging is that your week is typically packed. I still work 50%, and then I go to physiotherapy twice a week and speech therapy once a week. In addition, there’s a lot to do at home, and you also need to rest. So, I’ve always been honest with both the speech therapy and physiotherapy about the fact that home exercises sometimes get neglected.“*

## Discussion

In relation to our aim, we found that access to pAHP was often delayed. Many patients hesitated to seek help due to fear of contagion and perceptions of primary care. When support was accessed this commonly occurred through via self-referral or of referral from a health care professional. Regarding experience, patients generally valued allied health input and reported feeling taken seriously. Primary Allied Health Professionals recognized a clear role for themselves and engaged despite potential personal risks. In terms of delivery, care was initially provided remotely (telephone/video) due to public health restrictions, with most professionals resuming some in-person treatment as circumstances allowed. These findings align with our phenomenologically informed focus on lived experience: both patients and pAHPs described navigating uncertainty, perceived risk and shifting boundaries of care, which shaped how they interpret their symptoms, their needs, and the role of allied health care during the early pandemic.

Most patients reported positive experiences with the primary allied health care they received, consistent with literature on literature on home-based allied health care can support improvements in participation, quality of life, physical functioning, and psychological well-being [14,9]. Our patients particularly valued the personal attention, emotional support, and practical help in managing limitations, specifically, the imbalance between available energy and their day-to-day demands and responsibilities. Despite positive experiences and signs of recovery, many patients continued to experience significant fatigue and limitations in daily activities for months after initial infection [15–21]. These findings illustrate how patients made sense of their symptoms and recovery processes over time, a central concern in phenomenologically informed research. The persistence of fatigue despite supportive care, shaped how participants interpreted their bodily capacities and the meaning of progress, underscoring the importance of validating lived experience even when biomedical indicators remain inconclusive.

Our results highlight the lack of evidence-based guidelines during the early stages of the pandemic, where pAHPs often had to rely on their clinical judgment and past experiences in the absence of established guidelines for this novel disease. The adaptive shift in treatment focus, from initially promoting exertion to balancing load and capacity, illustrates an adaptive, patient centered approach. This adaptive strategy is consistent with findings from other studies where allied health professionals emphasized individualized care plans, active listening to patient concerns, and gradual, stepwise rehabilitation [20,22–24]. From a phenomenologically informed perspective, this shift illustrates how both patients and pAHPs interpreted bodily signals, limits, and recovery trajectories over time. The co-construction of meaning around “pushing,” “resting,” and “capacity” shaped rehabilitation decisions, aligning with our constructivist analytic lens.

Adaptivity was also needed for the organization of care. Our study found that while pre-existing multidisciplinary collaboration teams often facilitated better coordination, the broader primary care landscape faced significant operational difficulties [25]. Home health agencies and private allied health practices experienced reduced patient referrals, shortages of personal protective equipment, and considerable financial strain, with private practices particularly vulnerable due to limited access to government funding and ambiguity about ‘essential’ services [7,26,27]. The rapid acceleration of telehealth and hybrid care models, while improving access and offering advantages such as time-saving and suitability for patients avoiding in-person contact, did not fully replace the necessity of in-person visits [7,28,29]. This reflects the experience of our pAHPs and patients who largely preferred physical treatment sessions.

From a constructivist perspective, these organizational challenges and shifting modes of care delivery shaped how professionals and patients interpreted what “appropriate” or “safe” care meant during the pandemic. The introduction of telehealth required renegotiation of expectations, roles, and communication practices, processes that were co-constructed within the constraints and affordances of the rapidly evolving context.

The reported lack of coordinated care, particularly for non-hospitalized patients who remained at home, resonates with other research where healthcare providers reported losing control over patient situations and where allied health services specifically experienced disruption [30]. The hesitation of patients to seek help, as observed in our study, was also reported in research exploring delayed primary care during the pandemic, where patients avoided healthcare and downplayed symptoms [31–34]. Nevertheless, the adaptability and resilience of home-based primary care during the pandemic, where it was able to manage outbreaks and ensure continued access to care, has also been demonstrated in various contexts [15,22]. Taken together, these findings demonstrate how patients and professionals navigated uncertainty, shifting responsibilities, and limited system-level coordination. The meanings participants attributed to their symptoms, risks, and care options shaped their decisions to delay or engage with care, reflecting processes of sense-making that align with our phenomenological and constructivist approach.

Our research has several strengths and limitations. A key strength of the study is its broad scope, combining the perspectives of non-hospitalized patients and pAHPs. This dual lens offers a holistic account of early‑pandemic primary allied health care, capturing both the experiences of those receiving care and the perspectives of those delivering it. Patients also provided rich, detailed accounts of symptom patterns, pacing, energy management and the organization of care, which deepened our understanding of facilitators and barriers to recovery. At the same time, several limitations must be acknowledged. First, our sample included a notable proportion of participants with professional backgrounds in healthcare. While their familiarity with clinical reasoning and healthcare systems helped them articulate their experiences clearly, their dual positioning as both patients and professionals may have shaped how they interpreted symptoms, accessed care, and evaluated the allied health support they received. This may limit transferability to patients without such backgrounds.

Patients who were also health professionals contributed an ‘insider’ viewpoint. They provided precise descriptions of symptoms, pacing, and energy management, and informed reflections on interprofessional work and care organization. These data enriched our understanding of facilitators and barriers to recovery. The dual perspective also introduces limitations. Transferability to patients without health‑professional backgrounds may be reduced. Some accounts may be framed by professional norms or jargon. Access pathways and advocacy skills may be atypical for this group. Constraints that non‑professionals experience as barriers may be normalized. Social desirability towards colleagues may influence reporting. We sought to mitigate these risks through purposeful variation in sampling, reflexive team discussions, attention to deviant cases, and maintaining a transparent audit trail. Future research should purposively recruit patients without health‑professional roles and from underserved groups to examine whether similar patterns of experience and recovery are observed.

Second, in selecting pAHPs, we aimed to include professionals who had treated at least three patients with COVID-19. While this ensured relevant experience, it may have introduced bias by favoring professionals who were more active, innovative, or centrally involved in early pandemic care. Their perspectives may not fully represent those of pAHPs with less exposure to COVID-19 cases.

Finally, most patients in our sample were from the southern part of the Netherlands, where the pandemic initially hit hardest. This regional concentration reflects the early spread of the virus but limits the generalizability of findings to other parts of the country. Additionally, the time gap between illness and interview may have introduced recall bias, although the intensity of the experience likely preserved key memories.

Considered collectively, these strengths and limitations underscore the importance of interpreting our findings as co-constructed accounts situated within a unique historical and social context, an interpretive stance aligned with our phenomenological and constructivist orientation.

### Implications for health care services and future research

This study offers several important implications for both current practice and future research. First, in the absence of clear diagnostic criteria or established guidelines, it is essential that healthcare professionals acknowledge and validate patients’ symptoms. Especially during the early stages of a novel health crisis, recognizing the “realness” of patients’ complaints, even when biomedical explanations are lacking, can foster trust and support timely engagement with care.

Second, the flexibility shown by primary allied health care professionals in adapting their treatment strategies demonstrates the potential of allied health care to respond effectively to emerging health challenges. The shift from standardized protocols to individualized, evolving care plans highlights the sector’s capacity for innovation and responsiveness. Continued investment in adaptive, evidence-informed care is essential to support professionals in future crises.

Third, the pandemic underscored the importance of strong interprofessional networks in organizing care under pressure. Where such collaborations were already in place, care was more coordinated and responsive. Strengthening these networks in routine practice will enhance preparedness and resilience in future health emergencies.

Finally, although the evidence for effective allied health care interventions in long COVID is expanding [5,21,35–38], further research is needed to determine which treatment strategies are most effective in mitigating persistent symptoms following SARS-CoV-2 infection. Longitudinal and comparative research is particularly needed to understand how different rehabilitation approaches interact with patients’ lived experiences, recovery trajectories, and personal contexts.

## Supporting information

S1 Appendix Interview guide for patients. (DOCX)

S2 Appendix Interview guide for primary allied health care professionals. (DOCX)
