# Supplementary material for: Allied health care in the early stages of the COVID-19 pandemic: A qualitative study on the perceptions of non-hospitalized patients and allied health professionals
Source: PLoS One. 2026 Jan 23;21(1):e0341308. doi: 10.1371/journal.pone.0341308 (PMC12829828; doi:10.1371/journal.pone.0341308)
Supplement: S1 Appendix — (DOCX) [file pone.0341308.s001.docx]

**S1 Appendix. Interview guide for patients**

This appendix provides the semi-structured interview guide used for data collection.

**1. Situation prior to COVID-19**

- Please describe your daily life before COVID-19 (work and home situation).
- Were you familiar with allied healthcare? If so, by whom and for what purpose?

**2. Grand tour question**

- Please describe your experience with COVID-19 (onset, course of symptoms, hospitalization, rehabilitation).

**3. Access and organization of allied healthcare**

- How did you access primary allied healthcare (referral, self-initiated)?
- Was the care part of a COVID-19 recovery program?
- Which professionals were involved and in what order?
- Was there collaboration between allied health professionals and did this add value?
- What were your expectations of primary care allied healthcare?

**4. Content and delivery of treatment**

- What treatment did you receive (movement advice, ADL management, nutritional guidance, disease education)?
- Was contact face-to-face or online (video/telephone/apps)?
- How many contacts did you have, and did this meet your needs?
- Did you miss anything or receive conflicting advice?

**5. Financing and access**

- Was the care reimbursed? Did deductible or supplementary insurance affect access?
- In case of recovery care: was 6 months long enough? If not reimbursed, to what extent did this hinder you?
- Were you referred in a timely manner?

**6. Contribution to recovery**

- To what extent did allied healthcare contribute to your recovery (fully/partially)?
- Current health status, quality of life (physical, psychological, social), persistent symptoms.

**7. Overall experiences and support**

- Did you experience fear?
- Did you have non-allied healthcare needs?
- Did you receive formal/informal support or participate in peer groups?
- How did this influence your experience of illness and care?

**8. Demographic data**

- Age, gender, region, date of infection.

**9. Closing remarks**

- Anything else you would like to share.
