# Supplementary material for: Allied health care in the early stages of the COVID-19 pandemic: A qualitative study on the perceptions of non-hospitalized patients and allied health professionals
Source: PLoS One. 2026 Jan 23;21(1):e0341308. doi: 10.1371/journal.pone.0341308 (PMC12829828; doi:10.1371/journal.pone.0341308)
Supplement: S2 Appendix — (DOCX) [file pone.0341308.s002.docx]

**S2 Appendix. Interview guide for allied healthcare providers**

This appendix provides the semi-structured interview guide used for data collection.

**1. Grand tour question**

- Please describe your involvement with patients with COVID-19.

**2. Referrals and care pathways**

- Referral sources (specialists, GPs, fellow AHPs, self-referral).
- Timeliness and sufficiency of referrals; familiarity with allied professions; specificity of referral questions.

**3. Expertise and preparedness**

- Were you equipped for COVID-19 treatment? What specific expertise was needed and how was it acquired (workplace, association, other sources)?

**4. Organization and delivery of treatment**

- Barriers and facilitators; treatment locations (practice, home).
- Use of technology (video, instruments, phone, apps) and differences from pre-COVID.
- Changes across waves and practices likely to remain post-pandemic.
- Use and purchase of protective equipment.

**5. Professional support and collaboration**

- Peer coaching and support; collaboration with hospital, GPs, other AHPs.
- Experience with mandatory multidisciplinary work under COVID reimbursement.
- Who maintains overview of treatments and symptom trajectories; information flow among AHPs; communication with referrers.

**6. Financing and reimbursement**

- Reimbursement for COVID recovery care (basic insurance, deductibles); differences in rates across insurers; role of expertise and specialization.
- Late/residual symptoms: timing constraints for care initiation; funding for returns and erratic recovery trajectories.

**7. Treatment content by discipline**

- Physiotherapy/exercise therapy: capacity, fatigue, pulmonary issues, pacing.
- Dietetics: weight, taste/smell problems, macronutrient intake, GI complaints.
- Occupational therapy: self-reliance, cognition, ADL, work.
- Speech and language therapy: swallowing/breathing difficulties, laryngeal tension, voice problems.

**8. Outcomes and evaluation**

- Measurement/evaluation with or without patient; recovery (full/partial); quality of life; persistent symptoms; satisfaction; missed elements; suggested improvements.

**9. Follow-up**

- Differences and similarities in treatment possibilities and experiences compared to the first treatments given.

**10. Demographic data**

- Age, gender, years of experience, education and recognized specializations, employment setting and region.

**11. Final remarks**

- Post-interview: permission for recontact; how and when results will be available; thank the participant.
